# Beginner’s mind

**DOI:** 10.1080/17571472.2017.1370768

**Published:** 2017-09-08

**Authors:** Louise Younie

**Affiliations:** Barts and The London School of Medicine and Dentistry, Queen Mary University of London, London, UK

**Keywords:** MeSH Art, communication ERIC thesaurus reflection, creativity, interpersonal communication

## Abstract

The concept of ‘beginner’s mind’ invites the expert medical professional to be present to their patients, remaining curious and responsive in the face of the individuality of illness. Each patient is a universe of unknowns, presenting with suffering which cannot always be classified with a diagnosis. Improvisation and openness may not just benefit our patients enduring their patient journeys but may also revive and reconnect us with our own humanity.

**Why this matters to me:**

My experience as cancer patient brought home to me the value of encountering a doctor who was present and able to listen and respond to me as an individual. It did not necessarily take longer, but was about an attitude of heart.

Like any professional, as GPs we are at risk of presumption and habitual thinking. Beginner’s mind, that is recapturing the openness and curiosity modelled to us by children, can powerfully transform the medical encounter, allowing space for emergence of patient perspective and doctor response.

**Key message:**

Embrace curiosity and learning into our expert clinical practice

We shall not cease from exploration, and the end of all our exploring will be to arrive where we started and know the place for the first time.

TS Elliot

I watch my 18 month old daughter, Annika, carefully taking the clothes out of a basket, bringing them to me and then sometimes putting them back. Next, it’s the apples from an abundant harvest that are brought one by one, or moved around the kitchen or placed lovingly inside my boots. She explores the apple’s surfaces poking and prodding the holes and irregularities. ‘Rotten’ she says and brings one to me for consideration. I watch her work, or should I say play, by which I mean improvisatory engagement in her own active creative exploration. She enters into ‘flow’, a state of absorption in her creative pursuit.

A precious silence descends over a group of medical students in one of our creative workshops. They are concentrating hard as they explore ideas and lived experiences through paints, clay, material, feather, buttons etc. This is flow.

The idea of flow has been conceptualised, researched and written about by the psychologist Mihaly Csikszentmihalyi [1]. Flow is the zone we enter when in creative process or exploration we lose all sense of time, of self, of outcomes, instead being fully immersed in our activity, responding to materials, textures and processes. In flow process is valued above end product or any extrinsic good resulting from the work.

In her state of flow Annika is attentive to all that emerges from her enquiry with different materials as she seeks to make sense of a world that is unknown to her. She acts like a scientist doing experiments [2], bringing the whole of herself to bear on a situation or encounter, remaining present and alert. She comes with literally a beginner’s mind, a concept drawn from Zen Buddhism around bringing openness and curiosity to our experiences rather than leaning on a multitude of preconceived ideas [3].

A new medical student meeting her first patient on a home visit also brings a beginners perspective, illustrated by her openness and presence [4,5]. Her words can serve as an evocative reminder to us of our first experiences in medicine. In this first stanza of an arts-based reflective assignment, Georgina writes about her encounter with a lady suffering terminal cancer:

I am a canvas,
As blank as can be,
Inexperienced in suffering,
Ill-health still a mystery to me,
I sit waiting for your paintbrush,
For the colours to unveil,
I sit, I wait.
Georgina Maguire (2010) [6]

Returning to the busy surgery, our desire for expediency, our training in pattern recognition and our trust in learned ‘objective knowledge’ above the patient subjective experience may lead to dismissal of our patients’ lived experiences. As GP and cancer patient I have had an uncomfortable seat on both sides of the fence. ‘This chemotherapy does not cause palpitations’ I was told, whilst my heart raced up to 120 bpm at rest for days after my infusion. An acknowledgement of my symptoms possibly alongside the recognition, in this case, of medical impotence in terms of bringing down my heart rate, would not have changed my symptoms but probably the degree of suffering.

Despite the vast amount of learning about disease that doctors go through, the individuality and expression of illness often defies our clinical acumen. Each person with their unique character, life experiences and genetics is a universe unknown to the doctor. We join the dots in a consultation often with very few co-ordinates. This is a necessity, but sometimes we get it wrong. Being able to enter into ‘flow’ with a patient, being ‘in the zone’, listening, improvising a fresh response rather than trotting out standard phrases can bring about revelation, learning and change for the doctor and patient alike. Broyard [7], literary critic and editor of the New York Times book review, who died of prostate cancer in October 1990, suggests it is not just the patient who has something to gain when the doctor engages more fully and imaginatively with their patients:

Not every patient can be saved, but his illness may be eased by the way the doctor responds to him—and in responding to him the doctor may save himself. But first he must become a student again; he has to dissect the cadaver of his professional persona…by letting the sick man into his heart…they can share, as few others can, the wonder, terror, and exaltation of being on the edge of being, between the natural and the supernatural. [p. 57]

Of course, primary care with its standard of ten minute consultations is not terribly conducive to losing awareness of time and space as we connect with our patients. On the other hand, my cancer surgeon, for example, evidenced the capacity to remain curious and interested in his patients despite the pressures of the NHS. I will not forget the interest he took on his morning ward round in a fellow patients’ button which had been handmade by her grandmother. It was now sown onto the nighty she was wearing post-mastectomy for ease of access.

Remaining curious and open tie in with an *inductive approach* to consulting described as starting where the patient is at and with what they want to say, as opposed to the *deductive approach,* working to confirm or refute hypotheses in the clinical encounter. Fairhurst and May [8] have researched general practice consultations regarding inductive or deductive approaches and they found that an inductive approach allowed the patient to be part of the problem formulation which would then emerge over the length of the consultation. This was deemed good for the patient, as GPs who consulted more inductively had a greater sense of ‘knowing’ their patient. But the doctors working inductively also seemed to benefit, being found to have greater satisfaction in their practice.

The inductive approach to consulting involves improvisation and presence from the doctor as well as engagement with the individuality of the patient’s unique illness narrative. As Atluru, a doctor working in the Emergency Department writes in support of improvisation in medical education, improvisation is more about reacting, than acting [9]. It is about spontaneity and honesty in the clinical encounter [10]. I hold enormous gratitude to my cancer surgeon who recognised the particular suffering of a medic, with medical knowledge and access to the literature, facing all the uncertainties of cancer. He used research, story telling and case studies carefully chosen to resonate with me as a clinician to offer me hope in my darkest hour. He acted upon my concerns, for example, of lymph node swelling after mastectomy and delayed the radiotherapy to do an axillary clearance, acknowledging that I would be anxiously examining myself if ever there was any swelling there.

I began with the TS Elliot quote ‘we shall not cease from exploration, and the end of all our exploring will be to arrive where we started and know the place for the first time’. This, to me, encapsulates what is required for true expert clinical practice. As I recently shared with second year medical students, go on your learning journeys, become more confident and competent in medicine and then as a professional go back to what you had at the start of your medical studies. Recall when you first met patients, that quality of attention, the humility of mind and bring this perspective to your encounters whilst holding onto and using your tools and skills of your profession.

## Disclosure statement

No potential conflict of interest was reported by the author.
